# Curcumin Nanoencapsulated in Chitosan-Coated Liposomes: Therapeutic Potential against Pathogenic *Escherichia coli* and *Leishmania* spp.

**DOI:** 10.1021/acsomega.6c00783

**Published:** 2026-06-18

**Authors:** Jaqueline Barbosa de Souza, Jeferson Ricardo da Silva, Davi de Lacerda Coriolano, Luan Cícero da Silva, Policarpo Ademar Sales Júnior, Valéria Rego Alves Pereira, Luís André de Almeida Campos, Isabella Macário Ferro Cavalcanti

**Affiliations:** † Keizo Asami Institute (iLIKA), 28116 Federal University of Pernambuco (UFPE), Recife, Pernambuco 50670-901, Brazil; ‡ Aggeu Magalhães Institute (IAM/FIOCRUZ), Federal University of Pernambuco (UFPE), Recife, Pernambuco 50670-901, Brazil; § University of Pernambuco (UPE), Ouricuri, Pernambuco 50670-901, Brazil; ∥ Academic Center of Vitória (CAV), Federal University of Pernambuco (UFPE), Vitória de Santo Antão, Pernanmbuco 50670-901, Brazil

## Abstract

Infectious diseases caused by bacteria and protozoa continue to pose a critical public health challenge, exacerbated by the increasing resistance to conventional treatments. Curcumin, a broad-spectrum polyphenol with antimicrobial and antiparasitic properties, nevertheless presents clinical limitations associated with low solubility, chemical instability, and reduced bioavailability. In this study, curcumin nanoencapsulated into chitosan-coated liposomes (Lipo-CUR-Chi) was developed and characterized to evaluate its physicochemical performance, stability, antibacterial and antibiofilm activity against *Escherichia coli* strains, and antiparasitic efficacy against *Leishmania infantum* and *L*. *amazonensis*. The formulation showed high encapsulation efficiency (97.2%), stability for up to 120 days under refrigeration, and a marked increase in antimicrobial activity compared to free curcumin. A significant reduction in minimum inhibitory concentrations against *E*. *coli* was observed, along with a dose-dependent effect on biofilm formation inhibition. In RAW 264.7 macrophages, Lipo-CUR-Chi demonstrated lower cytotoxicity, indicating higher safety compared to free curcumin. In assays against *Leishmania* spp., CUR encapsulation resulted in increased potency and selectivity, particularly against *L*. *amazonensis*, approaching the therapeutic profile of amphotericin B but with lower toxicity. These results demonstrate that chitosan-coated liposomes are a promising platform to enhance the biological activity of curcumin, representing an innovative strategy for the treatment of bacterial and parasitic infections.

## Graphical abstract

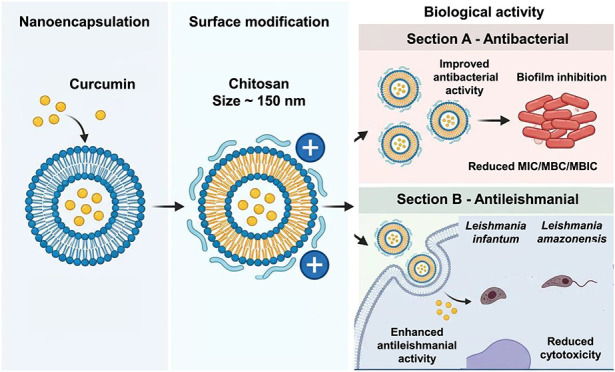

## 1 Introduction

Infectious diseases continue to represent one of the major challenges to global public health, being responsible for high morbidity and mortality, especially in tropical and subtropical countries. Among the microorganisms of greatest clinical relevance are certain bacteria and protozoa, whose resistance to conventional therapies and biological complexity hinder effective treatment.^1,2^

*Escherichia coli* is a Gram-negative bacterium that is a normal component of the intestinal microbiota; however, pathogenic strains are associated with a variety of infections, including gastroenteritis, urinary tract infections, septicemia, and neonatal meningitis.^3,4^ The emergence of multidrug resistant strains makes the development of new therapeutic approaches increasingly urgent, highlighting the need for alternative antimicrobial agents with innovative mechanisms of action.^5^

Similarly, protozoa of the genus *Leishmania* are responsible for leishmaniasis, an endemic disease in several regions of the world, which manifests in different clinical forms depending on the species involved and the host immune response.^6^ *L*. *infantum* is the etiological agent of visceral leishmaniasis, a potentially fatal disease characterized by prolonged fever, hepatosplenomegaly, and pancytopenia, whereas *L*. *amazonensis* is frequently associated with cutaneous leishmaniasis, causing chronic skin lesions that are difficult to heal and can progress to more severe forms in immunocompromised individuals.^7,8^

The life cycle of these parasites is complex, alternating between extracellular promastigote forms in the sandfly vector and intracellular amastigote forms in human macrophages. The intracellular amastigote is the clinically relevant target for chemotherapy, whereas promastigote assays are widely used as an initial screening model to identify compounds with leishmanicidal potential prior to evaluation in intracellular systems.^9^

In this context, bioactive natural compounds such as curcumin have attracted considerable interest due to their broad spectrum of pharmacological activities, including antimicrobial, antiparasitic, anti-inflammatory, and antioxidant effects.^10^ Curcumin, a polyphenol derived from the rhizome of *Curcuma longa*, shows promising potential in combating bacterial and parasitic infections.^11^ However, its direct clinical application is limited by low water solubility, chemical instability, rapid metabolism, and reduced bioavailability, factors that compromise therapeutic efficacy in both *in vitro* and *in vivo* models.^12^

Pharmaceutical nanotechnology has thus emerged as a promising tool to overcome these limitations. Encapsulation of curcumin in chitosan-coated liposomes offers an innovative strategy, combining protection of the active molecule, controlled release, and enhanced interaction with cellular membranes.^13^ Coating with chitosan, a biocompatible and biodegradable polysaccharide, not only improves liposome stability but also confers additional antimicrobial and mucoadhesive properties, enhancing curcumin delivery and efficacy.^14^ This nanotechnological approach allows for lower drug doses, reducing adverse effects while increasing therapeutic selectivity.^15^

In light of this context, the present study evaluated the therapeutic potential of curcumin nanoencapsulated in chitosan-coated liposomes against *E*. *coli* and the promastigote and amastigote forms of *L*. *infantum* and *L*. *amazonensis*, using parasite strains genetically modified to express β-galactosidase, which enables accurate quantitative assessment of antiparasitic activity.^16^ The chitosan-coated liposomal system was primarily designed for topical and dermal administration, particularly targeting cutaneous leishmaniasis caused by *L*. *amazonensis*, where localized delivery may help reduce systemic toxicity. Moreover, the physicochemical properties of the formulation indicate potential applicability for oral antibacterial therapy, although this route was not explored in the present study and remains a perspective for future investigation.

## 2 Methodology

### 2.1 Preparation of Chitosan-coated Liposomes Containing Curcumin

Liposomes containing CUR were prepared by lipid film hydration followed by sonication. Liposomes were composed of soybean phosphatidylcholine (Lipoid S100), cholesterol and Tween 80 at a molar ratio of 7:2:1. Curcumin and lipophilic components were dissolved in a chloroform:methanol mixture (2:1, v/v) to form a homogeneous organic phase under magnetic stirring prior to lipid film formation. Tween 80 was incorporated as an anionic surfactant to improve curcumin solubilization and promote vesicle stability by reducing lipid aggregation during liposome formation.

Subsequently, the lipid film was formed by evaporation of the solvents under reduced pressure, which was subsequently resuspended in phosphate buffer solution (pH = 7.4), spontaneously forming large multilamellar vesicles were sonicated using a probe sonicator at 40% amplitude for 5 min (cycles of 30s on/30s off) under ice bath cooling to obtain small unilamellar vesicles.^17^

After that, the chitosan was solubilized in glacial acetic acid. The 1% chitosan solution was subjected to constant stirring overnight. The liposomes were added dropwise into the chitosan solution and kept under magnetic stirring for 1 h to obtain Lipo-CUR-Chi.^18^

### 2.2 Characterization of Liposomes

Lipo-CUR-Chi were subjected to physicochemical characterization through analysis of particle size (Ø), polydispersity index (PDI), zeta potential (ζ), and pH as previously described by Souza et al.^17^ Liposome dispersions were sized by photon correlation spectroscopy using a Zetasizer Nano-ZS90 (Malvern, Worcestershire, UK).

For particle size and zeta potential measurements, liposomal dispersions were diluted at a ratio of 1:50 (v/v) in purified water prior to analysis. Measurements were performed at 25 °C with a fixed 90° angle, and the results were expressed as the mean hydrodynamic diameter of the liposomes (nm). Liposome zeta potential was measured after diluting the liposome dispersion in an ultrapure water solution. Liposome surface charge (mV) was assessed using a Zetasizer Nano-ZS90 (Malvern, Worcestershire, UK). Liposome pH was measured with a glass electrode and a MS Tecnopon digital pH meter (mPA-210P, São Paulo, Brazil) at room temperature.

### 2.3 Determination of the Content and Encapsulation Efficiency of Lipo-CUR-Chi

To determine the CUR content in the formulations, the liposomes were diluted in CH_3_OH, centrifuged for 10 min, and the supernatant was measured spectrophotometrically at a wavelength of 420 nm. The results were expressed as a percentage of the mean absorbance. The experiment was performed in triplicate in three independent experiments.

The encapsulation efficiency (EE%) of CUR was determined by ultrafiltration/ultracentrifugation and was performed at 7500*g* for 60 min at 4 °C using Amicon Ultra centrifugal filters (Amicon Ultra Centrifugal Filters; Millipore, Billerica, MA). Liposome samples were inserted into the filters and subjected to ultracentrifugation at 8,000 rpm at 4 °C for 1 h. An aliquot of the filtered sample was diluted in CH_3_OH. The EE% of CUR was measured spectrophotometrically, and the results were expressed as a percentage of the mean absorbance.

Curcumin quantification was performed using a calibration curve constructed in methanol at concentrations ranging from 1 to 20 μg/mL (*R* ^2^ > 0.99). Encapsulation efficiency was calculated based on the quantified curcumin concentration rather than mean absorbance values. Drug encapsulation efficiency data were calculated using the equation described below

%EE=total CUR−CUR filtrate×100/total CUR

### 2.4 Stability of Liposomal Dispersions

The physicochemical stability of Lipo-CUR-Chi dispersions was evaluated 24 h after formulation, after storage in a refrigerator at 2 °C. The formulations were monitored after 7, 15, 30, 60, and 120 days after formulation by evaluating the following parameters: macroscopic appearance, Ø, PDI, ζ, pH, content and encapsulation rate for the drug-containing liposomes.

### 2.5 Evaluation of the Antibacterial Activity of Lipo-CUR-Chi

The *in vitro* antibacterial activity of curcumin (CUR) and Lipo-CUR-Chi was evaluated using the broth microdilution method according to the Clinical and Laboratory Standards Institute guidelines.^20^ Tobramycin (TOB) was included as a positive control, and the Lipo-Chi formulation was also evaluated to assess the effect of the carrier system. Initially, Müeller-Hinton broth was distributed into each well of the plates. All formulations were prepared under aseptic conditions and sterilized by filtration through 0.22 μm membrane filters prior to biological assays to avoid microbial contamination. Subsequently, CUR and Lipo-CUR-Chi were added through serial dilution, and finally, suspensions of *Escherichia coli* ATCC 25922, *E*. *coli* NCTC13846, and *E*. *coli* H10407 were added. The microplates were incubated at 35 °C for 24 h, and the minimum inhibitory concentration (MIC) was determined spectrophotometrically at a wavelength of 630 nm, and the absorbance at 630 nm was used as an indirect measure of bacterial growth.

The minimum bactericidal concentration (MBC) was determined after the MIC results. An aliquot of microorganisms from wells showing no visible growth was inoculated on to Müeller-Hinton agar, and the plates were incubated at 35 °C for 24 h. After this period, the MBC was determined as the lowest concentration at which no microbial growth was observed.^20^ The entire experiment was performed in independent triplicates.

### 2.6 Evaluation of the Antibiofilm Activity of Lipo-CUR-Chi

The antibiofilm activity of CUR, Lipo-Chi and Lipo-CUR-Chi against *E*. *coli* ATCC H10407 was determined using the crystal violet method. Initially, the bacterial strain was adjusted to the 0.5 McFarland scale. Cell counts were confirmed by spectrophotometry at 630 nm, and the bacterial suspensions were distributed onto flat-bottom microdilution plates to a final concentration of 10^5^ CFU/mL and incubated at 35 °C for 24 h. After biofilm growth, the contents of each well were aspirated, and serial dilutions of CUR and Lipo-CUR-Chi were prepared in tryptic soy broth (TSB) and added to each well.

The plates were again incubated at 35 ± 2 °C for 24 h. After incubation, the well contents were aspirated, and washes were performed with saline (0.9%). The plates were dried, and the adhered bacteria were then fixed with 99% methanol. After fixation, the methanol was removed, and the plates were allowed to dry again.

Subsequently, the bacteria adhered to the plates were stained with 1% crystal violet. Excess dye was removed, and each well was washed with saline. The results were then analyzed using spectrophotometry at 570 nm (Multiskan FC microplate photometer, Thermo Scientific, Madrid, Spain). The Minimum Biofilm Eradication Concentration (MBEC) was determined as the lowest concentration capable of inhibiting biofilm formation.^21^ The entire experiment was performed in independent triplicates.

### 2.7 Cytotoxicity in RAW 264.7 Cells

RAW 264.7 cells were used to assess cellular cytotoxicity by the MTT assay. Cells (0.2 × 10^5^ cells/well) were seeded in 96-well plates and incubated for 24 h at 37 °C in 5% CO_2_ for adhesion. Compounds were added at eight concentrations and incubated for 48 h. Amphotericin B, CUR, and Lipo-CUR-Chi had their cytotoxicity assessed over different concentration ranges: 40–0.31 μg/mL for Amphotericin B and 100–0.78 μg/mL for CUR and Lipo-CUR-Chi.

After incubation with the compounds, 3-[4,5-dimethylthiazol-2-yl]-2,5-diphenyltetrazolium bromide (MTT) was added at 5 mg/mL in PBS. The cell culture medium was discarted after 2 h, and 100 μL of DMSO was added to solubilize the formazan crystals. Absorbance was measured at 570 nm. The cytotoxic concentration that inhibits 50% of cells (CC_50_) was determined by nonlinear regression from the eight duplicate concentrations using GraphPad Prism 8.0 software (GraphPad Software, San Diego, CA). Two independent experiments were performed.

### 2.8 *In Vitro* Activity in Promastigotes

Promastigote forms of *L*. *amazonensis* (strain WHOM/00-LTB0016) and *L*. *infantum* (strain MHOM/MA/67/ITMAP-263) that express the β-galactosidase gene from *E*. *coli* ^16^ were maintained at 26 °C in Schneider’s medium (Sigma) supplemented with 10% fetal bovine serum (complete medium) and hemin (2.5 μg/mL). Parasites in the exponential growth phase were used in all experiments. For the leishmanicidal activity assay, the parasites were counted and diluted in complete Schneider’s medium (Sigma) to 1 × 10^6^ promastigotes/mL (0.1 × 10^5^ promastigotes per well).

The parasites were incubated at 26 °C in the presence of different concentrations of CUR and Lipo-CUR-Chi (100–0.78 μg/mL) and Amphotericin B (10–0.078 μg/mL) for 72 h. Parasites incubated with culture medium only and culture medium without parasites containing the compounds were used as controls. After incubation, CPRG solution (500 μM, 0.5% Nonidet P-40, in PBS) was added, followed by a new incubation for 10 min at 22 °C. The absorbance was read at 570 nm on the THERMO SCIENTIFIC Multiskan FC spectrophotometer. The leishmanicidal activity of the compounds was evaluated by the decrease in β-galactosidase activity in treated cultures compared to the untreated control culture. Miltefosine was used as a positive control. IC_50_ values were calculated by nonlinear regression analysis using GraphPad Prism software. Each assay was performed in duplicate.

The selectivity index (SI) was calculated by the ratio between CC_50_ and IC_50_, serving as an initial parameter to evaluate the selectivity of the compound against the promastigote forms of *Leishmania* sp.

### 2.9 Tests with Amastigote Forms of *Leishmania* spp

The promastigote forms of *L*. *infantum* (strain MHOM/MA/67/ITMAP-263) and *L*. *amazonensis* (strain WHOM/00 LTB 0016) expressing the β-galactosidase gene^16^ were cultured in Schneider medium with 10% fetal bovine serum, 2.5 μg/mL hemin, 1% antibiotics (100 IU/mL penicillin and 100 μg/mL streptomycin), and 50 μg/mL hygromycin, at 26 °C. To obtain the amastigote forms, RAW 264.7 cells were seeded (0.2 × 10^5^ cells/well) in 96-well plates and allowed to adhere for 24 h at 37 °C in 5% CO_2_.

The adhered cells were then infected with stationary-phase promastigotes at a ratio of 1:15 at 37 °C for 6 h. Noninternalized parasites were then washed away, and the infected cultures were incubated for 24 h in RPMI 1640 complete medium (negative control) and treated with the compounds (0.78 to 100 μg/mL). Amphotericin B (10–0.078 μg/mL) was used as positive controls.

After 24 h, the cultures were washed again, the medium was replaced, and 100 μM chlorophenol red β-d-galactopyranoside (CPRG) and 0.1% Nonidet P-40 were added to the plates, which were incubated for 2 to 6 h at 37 °C. Absorbance was measured at 570 nm in an automated microplate reader. The results are expressed as percentage of parasite growth inhibition. The IC_50_ was determined by nonlinear regression from eight duplicate concentrations, using GraphPad Prism 8.0 software. The selectivity index (SI) was calculated by the ratio between CC_50_ and IC_50_, serving as an initial parameter to evaluate the selectivity of the compound against the amastigote forms of *Leishmania* sp.

### 2.10 Statistical Analyses

All experiments were performed in at least three independent replicates. Statistical analysis was conducted using the nonparametric Mann–Whitney test for pairwise comparisons and two-way analysis of variance (ANOVA) followed by Bonferroni’s multiple comparisons post hoc test for multiple group analyses. Differences were considered statistically significant when *p* < 0.05.

## 3 Results and Discussion

### 3.1 Physicochemical Characterization

The Chitosan-coated liposomes (Lipo-Chi) showed a Ø of 97.72 ± 0.70 nm, a PDI of 0.298 ± 0.100, a ζ-potential of +12.40 ± 0.90 mV, and pH 5.0. The Lipo-CUR-Chi exhibited Ø of 142.03 ± 2.24 nm, PDI of 0.386 ± 0.22, ζ of +15.0 ± 0.66 mV and pH 5.1, as described in Table 1. The Lipo-Chi was developed exclusively for methodological purposes, serving as a control to evaluate the encapsulation of curcumin within chitosan-coated liposomes.

**Table 1:** Characterization of Lipo-Chi and Lipo-CUR-Chi^a^

formulation	Ø (nm)	PDI	ζ (mV)	pH	content	%EE
Lipo-Chi	97.72 ± 0.70	0.298 ± 0.10	+12.40 ± 0.93	5.0		
Lipo-CUR-Chi	142.03 ± 2.24	0.386 ± 0.22	+15.0 ± 0.66	5.1	99.90 ± 0.10%	97.20 ± 0.62%

a Ø: Particle size; PDI: Polydispersity index; ζ: Zeta potential; %EE: Encapsulation efficiency; Lipo-Chi: Chitosan-coated liposomes; Lipo-CUR-Chi: Chitosan-coated liposomes encapsulating CUR.

The physicochemical characterization of the Lipo-Chi and Lipo-CUR-Chi formulations revealed an increase in particle diameter following curcumin incorporation, from approximately 100 to 150 nm, this size increase is consistent with drug loading within the lipid bilayer. Although an increase in PDI was also observed, the values remained within an acceptable range for topical and dermal delivery systems. As uncoated liposomes were not evaluated, no direct conclusions regarding the isolated effect of chitosan coating on vesicle properties were drawn.^17^

The PDI showed a slight increase, from 0.298 ± 0.10 to 0.386 ± 0.22, remaining within acceptable limits for pharmaceutical applications. These values indicate that the particle size distribution remains adequate, ensuring stability and functionality of the nanostructures.^22,23^

The zeta potential of the liposomes were +12.4 ± 0.93 mV and +15.0 ± 0.66 mV, reflecting the presence of protonated amine groups from chitosan on the liposome surface. This charge confirms the effectiveness of the coating, with chitosan performing its expected role in providing colloidal stability and favoring electrostatic interactions with cell membranes, without causing significant alterations to the particles.^24,25^

The pH of the formulations remained around 5.0, indicating that the encapsulation process did not significantly alter the acidity of the samples. This slightly acidic pH is favorable for curcumin stability during gastrointestinal transit, protecting the drug from degradation and allowing controlled release in the small intestine, which contributes to increased bioavailability and therapeutic efficacy.^17,26^

Curcumin encapsulation in chitosan-coated liposomes also proved to be highly efficient, with a drug content of 99.9 ± 0.10% and %EE of 97.2 ± 0.62%. These results indicate that almost all the added curcumin was incorporated into the liposomes, ensuring that the drug remains within a therapeutic range long enough to exert its biological effect. Nanoencapsulation may enhance curcumin’s aqueous solubility, facilitating its administration, absorption, and systemic distribution, potentially improving the therapeutic response against pathogenic microorganisms and parasites.^27,28^

Thus, these characteristics make the formulation promising for future therapeutic applications, offering an effective strategy to overcome limitations related to solubility, bioavailability, and drug stability, as well as enabling targeted action against clinically relevant bacteria and parasite.

### 3.2 Stability of Liposomal Dispersions

Table 2 presents the physicochemical stability parameters of the Lipo-CUR-Chi formulation stored at 2 °C for 120 days. A progressive increase in mean particle size (Ø) was observed, accompanied by a slight increase in PDI over time. Specifically, the initial diameter increased from 149.12 ± 0.50 nm to 169.44 ± 1.71 nm after 120 days. Similarly, the PDI gradually increased from 0.396 ± 0.12 to 0.436 ± 0.50, indicating a modest rise in vesicle population heterogeneity, although remaining within acceptable limits for liposomal systems. The zeta potential decreased from +14.62 ± 0.91 mV to +11.40 ± 0.74 mV, while maintaining positive values throughout the storage period. Additionally, the pH remained stable (from 5.2 to 5.0), suggesting no significant degradation of curcumin or the lipid matrix.

**Table 2:** Stability Study of Lipo-CUR-Chi at 2 °C^a^

formulation	time (days)	Ø (nm)	PDI	ζ (mV)	pH
Lipo-CUR-Chi	7	149.12 ± 0.50	0.396 ± 0.12	+14.62 ± 0.91	5.2
	14	156.42 ± 2.90	0.401 ± 0.31	+13.44 ± 1.64	5.0
	30	163.55 ± 1.56	0.412 ± 0.10	+12.61 ± 0.90	5.0
	60	166.03 ± 2.40	0.424 ± 0.16	+12.12 ± 0.64	5.0
	120	169.44 ± 1.71	0.436 ± 0.50	+11.40 ± 0.74	5.0

a Ø: Particle size; PDI: Polydispersity index; ζ: Zeta potential; %EE: Encapsulation efficiency; Lipo-CUR-Chi: Chitosan-coated liposomes encapsulating CUR.

The stability assessment of the formulations indicated that Lipo-CUR-Chi maintained adequate physicochemical parameters for therapeutic applications over 120 days at 2 °C, with no evidence of significant aggregation or degradation. The gradual increase in hydrodynamic diameter is expected in liposomal systems and may reflect fusion or reorganization processes of the lipid bilayer during storage.^29^ Despite this increase, the formulation remained within the nanometric scale, a desirable feature for therapeutic applications, particularly because it favors internalization into macrophages, a key factor when evaluating therapy for leishmaniasis, as it allows the drug to directly reach the host cells where the parasite resides.^30^

The PDI of Lipo-CUR-Chi showed slightly higher values, yet remained acceptable for nanoparticle dispersions, suggesting relative homogeneity. This behavior aligns with previous reports of polymer-coated liposomal systems, in which PDI increases over time reflect structural adaptations without critical loss of stability.^31^ Moreover, the moderate reduction in ζ values over time did not compromise colloidal stability, as the formulations maintained a positive charge above +9 mV, a level considered satisfactory for nanometric dispersions stabilized by polyelectrolytes.^32^

Regarding pH, the dispersions exhibited only minor variations, suggesting that curcumin remained stable within the liposomal environment during refrigerated storage. This is consistent with recent findings demonstrating that encapsulation in polymer-coated liposomes reduces oxidative and photochemical degradation of curcumin, preserving its bioactivity.^33^

Although only minor variations were observed in the mean particle size, the increase in PDI suggests greater heterogeneity in the vesicle population, possibly due to chitosan coating and intermolecular interactions. Despite this, Lipo-CUR-Chi exhibited satisfactory physicochemical stability over 120 days at 2 °C, as evidenced by the overall maintenance of its key parameters. These findings indicate that the formulation remains suitable for storage under refrigerated conditions, reinforcing its potential for pharmaceutical applications, particularly in the treatment of bacterial infections and leishmaniasis.

### 3.3 Antibacterial Activity

The antibacterial activity assessment revealed that free CUR exhibited strain-dependent variability in both MIC and MBC values among *E*. *coli* strains. Specifically, *E*. *coli* ATCC 25922 showed MIC and MBC values of 125 μg/mL, whereas *E*. *coli* NCTC 13846 demonstrated markedly reduced susceptibility, with both MIC and MBC reaching 1000 μg/mL. For *E*. *coli* H10407, intermediate sensitivity was observed, with a MIC of 250 μg/mL and an MBC of 500 μg/mL.

In contrast, Lipo-CUR-Chi displayed consistent antibacterial activity across all tested strains, with uniform MIC and MBC values of 62.5 μg/mL. This reduction was statistically significant when compared to free CUR (*p* < 0.05–0.0001), depending on the strain, highlighting the substantial enhancement in antimicrobial efficacy promoted by nanoencapsulation (Table 3). Notably, the empty liposomal formulation (Lipo-Chi) did not exhibit any inhibitory activity at any of the tested concentrations, confirming that the observed antibacterial effects are exclusively associated with the presence of curcumin in the formulation.

**Table 3:** Evaluation of the Antibacterial Activity of CUR and Lipo-CUR-Chi against *E*. *coli* ATCC 25922, NCTC 13846 and H10407^a^

	CUR		Lipo-Chi		Lipo-CUR-Chi		TOB	
	μg/mL							
Bacteria	MIC	MBC	MIC	MBC	MIC	MBC	MIC	MBC
*E*. *coli* ATCC 25922	125	125	ND	ND	62.5*	62.5	0.97	1.95
*E*. *coli* NCTC 13846	1000	1000	ND	ND	62.5****	62.5	1.95	7.80
*E*. *coli* H10407	250	500	ND	ND	62.5***	62.5	0.97	3.9

a CUR: Curcumin; Lipo-Chi: Chitosan-coated liposomes; Lipo-CUR-Chi: Chitosan-coated curcumin-containing liposome; MIC: Minimum inhibitory concentration; MBC: Minimum bactericidal concentration; ND: Not determined. Values represent mean of three independent experiments. Statistical analysis was performed using one-way ANOVA followed by Bonferroni’s post hoc test. Differences were considered statistically significant at *p* < 0.05. Significance levels were defined as *p < 0.05, **p < 0.01, ***p < 0.001, ****p < 0.0001.

Tobramycin (TOB) was included solely as a positive control for bacterial growth inhibition, exhibiting low MIC and MBC values consistent with its well-established antibacterial activity against *E*. *coli*. However, it was not the focus of comparative analysis, serving only to validate the experimental conditions.

Consistent with these findings, the concentration/response curves demonstrated that Lipo-CUR-Chi exerted significantly greater antibacterial activity than free CUR across all strains (Figure 1). In *E*. *coli* ATCC 25922 (Figure 1A), Lipo-CUR-Chi achieved near-complete growth inhibition (∼100%) at the highest concentration tested, whereas free CUR displayed a more gradual and less pronounced inhibitory profile.

**Figure 1:**
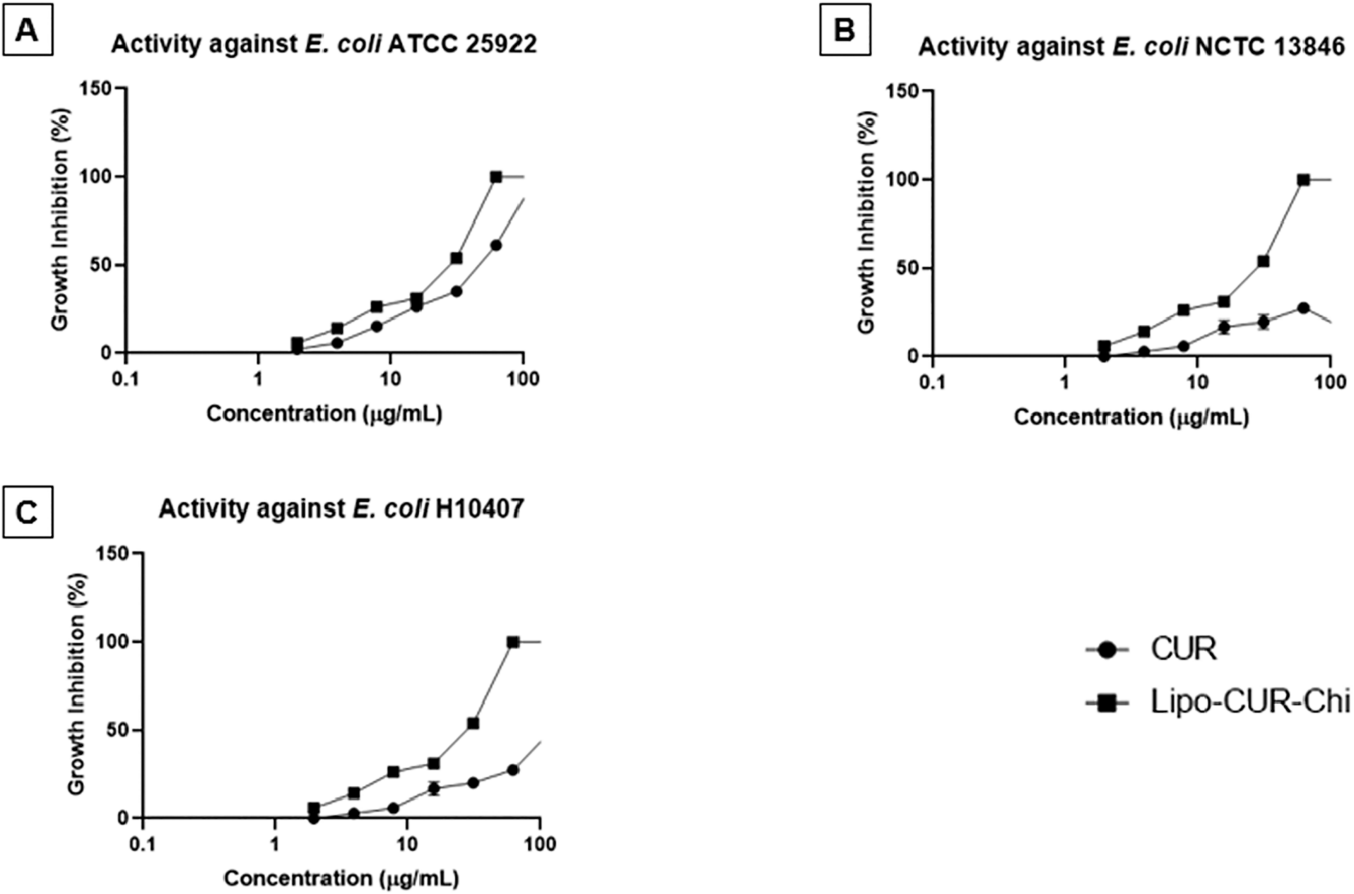
Antibacterial activity of CUR and Lipo-CUR-Chi against different *Escherichia coli* strains. Growth inhibition (%) was evaluated across increasing concentrations (μg/mL) against (A) *E*. *coli* ATCC 25922, (B) *E*. *coli* NCTC 13846, and (C) *E*. *coli* H10407. Data demonstrate a concentration-dependent increase in antibacterial activity for both formulations, with Lipo-CUR-Chi exhibiting enhanced efficacy compared to free CUR. Points represent mean values of independent experiments.

A similar pattern was observed for *E*. *coli* NCTC 13846 (Figure 1B), which exhibited pronounced resistance to free CUR, with minimal inhibition even at elevated concentrations. In contrast, Lipo-CUR-Chi maintained a strong inhibitory effect, indicating its ability to partially overcome intrinsic tolerance mechanisms in this strain. For *E*. *coli* H10407 (Figure 1C), both treatments showed limited activity at lower concentrations, however, Lipo-CUR-Chi again demonstrated superior efficacy, reaching complete inhibition at higher concentrations, while free CUR remained only partially effective.

The antibacterial activity of CUR against Gram-negative bacilli is naturally limited, as demonstrated by Kareem et al.,^34^ who reported MIC and MBC values of 256 and 512 μg/mL, respectively, against *Campylobacter jejuni* ATCC 33560. This low efficacy is associated with the difficulty of CUR permeating bacterial membranes due to its lipophilic nature, which reduces its ability to inhibit microbial growth. In this context, nanostructured systems emerge as strategic tools to overcome these limitations, enabling targeted delivery of the drug directly into bacterial cells, increasing local compound concentration, and enhancing antimicrobial action.^33^

Studies using CUR encapsulated in nanosystems have shown a significant reduction in inhibitory concentrations against Gram-negative bacteria, demonstrating the potentiating effect of nanoencapsulation. Targhi et al.^35^ observed that CUR encapsulated in niosomes (Cur-Nio) had an MIC of 25 μg/mL against clinical isolates of *Pseudomonas aeruginosa*, whereas free CUR exhibited an MIC of 50 μg/mL, indicating that delivery via nanostructured systems amplifies the compound’s efficacy.

Similarly, Chen et al.^13^ evaluated the antimicrobial activity of nanofibers composed of liposomes containing CUR and observed enhanced efficacy against *E*. *coli*, with an inhibition zone of 38.0 ± 1.1 mm, higher than that observed for free CUR (32.4 ± 0.2 mm). These findings reinforce the potential of nanostructured systems to increase local availability of CUR, promoting greater interaction with the bacterial membrane and consequently enhancing its antibacterial activity.

In this context, the results obtained in the present study confirm this effect, showing that Lipo-CUR-Chi significantly reduced MIC and MBC values against various *E*. *coli* strains compared to free CUR. The potentiation of antibacterial activity is related to the presence of curcuminoids, the main secondary metabolites of CUR, which exhibit action against both susceptible bacteria and strains resistant to conventional antibiotics.^36^

Therefore, these findings highlight that CUR nanoencapsulation not only improves its solubility and bioavailability but also maximizes its biological activity, emphasizing chitosan-coated liposomes as promising controlled-release systems capable of overcoming bacterial barriers and enhancing therapeutic efficacy against resistant pathogens.

A limitation of this study is the absence of a reference antibacterial drug tested under identical experimental conditions, which should be addressed in future investigations to allow direct comparison of efficacy.

### 3.4 Antibiofilm Activity

Biofilm inhibition showed a dose-dependent response for the different agents tested. The greatest inhibition was observed at the concentration corresponding to the MIC, while the lowest occurred at the MIC/16, as illustrated in Figure 2. The results demonstrated significant variation in biofilm inhibition levels, with CUR presenting inhibition between 36.3% and 71%, and the Lipo-CUR-Chi formulation between 26.61% and 85.07%. As expected, the Lipo-Chi formulation showed no effect on biofilm formation at any of the concentrations evaluated. These findings highlight the importance of agent concentration in the efficacy of biofilm inhibition, providing relevant information for the development of therapeutic strategies and future clinical applications.^37^

**Figure 2:**
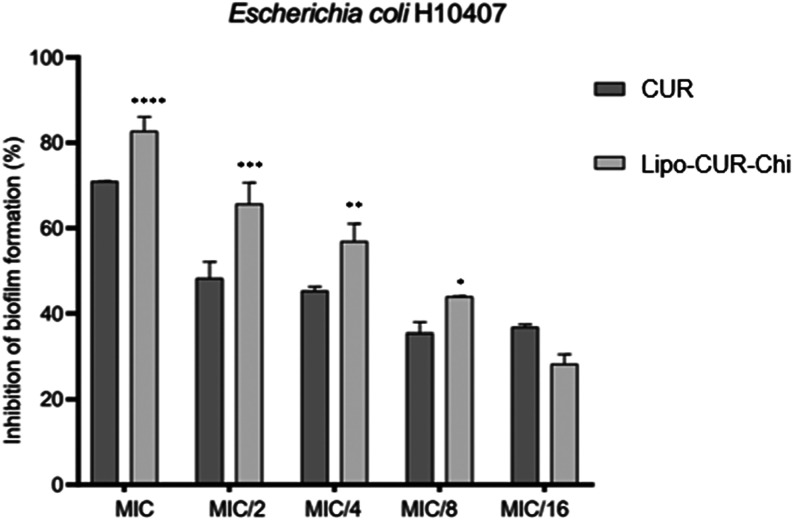
Biofilm inhibition of *E*. *coli* H10407 after treatment with curcumin-loaded chitosan-coated liposomes. Data were analyzed using two-way analysis of variance (ANOVA) followed by Bonferroni’s multiple comparisons post hoc test. Differences were considered statistically significant when *p < 0.05, **p < 0.01, ***p < 0.001, ****p < 0.0001.

Biofilms are microbial communities organized within an exopolysaccharide matrix that acts as a protective barrier, isolating bacteria from antimicrobial agents. For a compound to be effective in disrupting biofilms, it must be able to penetrate this matrix and eliminate the bacteria within these clusters.^38,39^ In this context, the biofilm inhibition activity observed in the formulations developed in this study can be attributed to the action of curcumin-encapsulating liposomes, which show potential in prevention, particularly during the adhesion phase of these complex bacterial structures.^40^

Biofilm formation begins with bacterial adhesion to a surface, followed by cell growth and exopolysaccharide production, processes regulated by quorum sensing (QS), which coordinates bacterial behavior according to population density. Bacterial adhesion to surfaces and exopolysaccharide production are critical for biofilm consolidation. Strategies for inhibiting biofilm formation include direct attacks on bacterial cells, prevention of surface adhesion, or disruption of QS.^41^

Studies have shown that CUR encapsulation in liposomes enhances antibiofilm efficacy. Shariffian et al.^42^ demonstrated that curcumin nanoparticles (Nano-Cur) significantly reduced biofilm formation by *P*. *aeruginosa* ATCC 10145. Without Nano-Cur, the strain produced robust biofilm, whereas with Nano-Cur at concentrations of 15 and 20 μg/mL, biofilm formation was reduced to moderate and weak levels, respectively, highlighting the potential of encapsulated curcumin in biofilm inhibition.

Similarly, Hu et al.^27^ evaluated *Streptococcus mutans* biofilm formation and observed a significant reduction in the group treated with curcumin-containing liposomes (Lipo-CUR) compared to free curcumin at 10 μM. Free curcumin lost its effect after 4 h, by which time the biofilm was already established, whereas encapsulated curcumin remained associated with the biofilm, exerting continuous antibacterial activity throughout the cultivation period.

In addition to the action of curcumin itself, the physicochemical properties of liposomes can enhance the antibiofilm effect. Studies indicate that liposomes with diameters between 100 and 300 nm favor penetration and targeted delivery of antibacterial agents within the biofilm. In this regard, the liposomes developed in this study had an average size of approximately 142 nm, suitable for promoting effective penetration and prolonged action of curcumin on bacteria within the biofilm.^17,43^

### 3.5 Evaluation of Cytotoxicity in RAW 264.7 Cells

The cell viability curves demonstrated a concentration-dependent reduction in RAW 264.7 macrophages for all treatments (Figure 3). Free CUR showed a pronounced cytotoxic effect, with a sharp decrease in viability as concentrations increased, indicating reduced cell tolerance. In contrast, Lipo-CUR-Chi exhibited a more gradual reduction in viability, maintaining higher cell survival across the tested concentrations. As expected, Lipo-Chi did not exhibit activity under the tested conditions.

**Figure 3:**
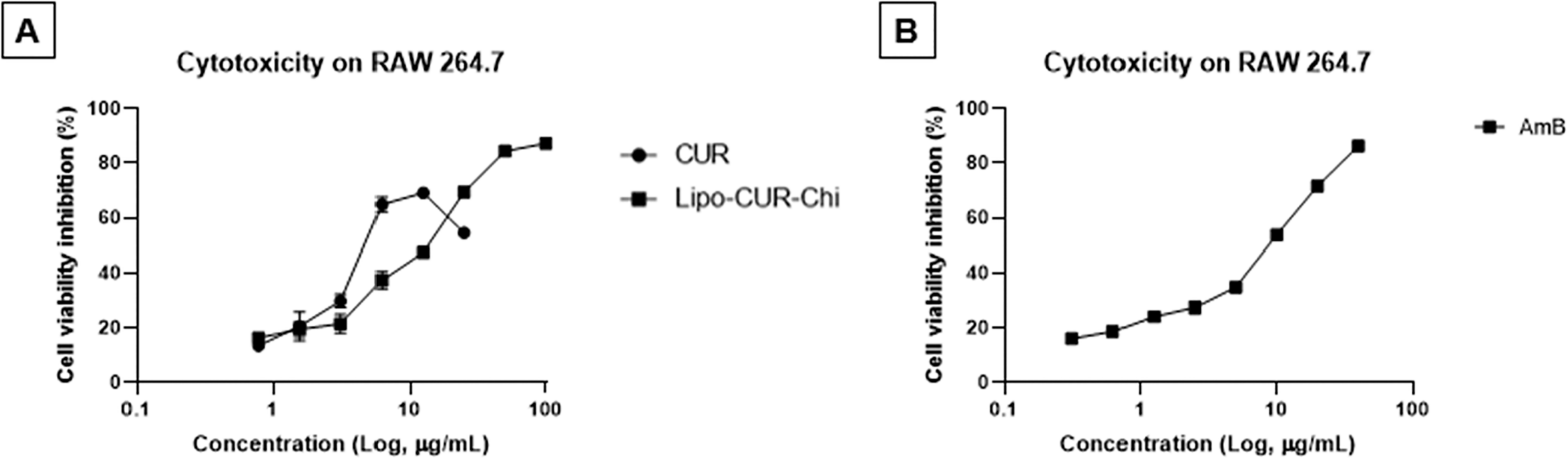
Cell viability curves of RAW 264.7 cells after treatment with different formulations. (A) Comparison between CUR and Lipo-CUR-Chi, showing a concentration-dependent decrease in cell viability (log scale, μg/mL), with greater cytotoxic effect observed for the encapsulated formulation at higher concentrations. (B) Cell viability curve for Amphotericin B (AmB), demonstrating a dose-dependent reduction in cell viability.

These findings are consistent with the CC_50_ values, where CUR presented a CC_50_ of 3.7 ± 0.1 μg/mL, indicating high cytotoxicity at low concentrations. Conversely, Lipo-CUR-Chi showed a higher CC_50_ (12.7 ± 0.1 μg/mL), suggesting an improved safety profile and reduced toxicity toward mammalian cells. Amphotericin B displayed an intermediate cytotoxic profile, with a CC_50_ of 8.5 μg/mL Table 4.

**Table 4:** Cytotoxicity of CUR and Lipo-CUR-Chi, as Well as the Reference Drug Amphotericin B, on RAW 264.7 Macrophages after 48 h of Treatment^a^

Compound	CC_50_ over RAW 264.7
μg/mL	
CUR	3.7 ± 0.1
Lipo-Chi	ND*
Lipo-CUR-Chi	12.7 ± 0.1
AmB	8.5 ± 0

a CC_50_: Cytotoxic concentration 50%; CUR: Curcumin; Lipo-Chi: Chitosan-coated liposomes; ND: Not determined; Lipo-CUR-Chi: Liposome containing curcumin coated with chitosan; AmB: Amphotericin B.

These findings align with well-described trends in the literature for nanosystems, in which encapsulation typically reduces cytotoxicity while simultaneously enhancing biological activity by improving solubility, stability, and controlled drug release.^44^ In RAW 264.7 macrophage cell models, nanostructured curcumin systems often do not exhibit significant cytotoxicity within the tested ranges, reinforcing the protective role of the carrier compared to free curcumin.^45,46^

Chitosan coating emerges as a decisive factor. When comparing Lipo-CUR-Chi with free CUR, there is a progressive increase in CC_50_, resulting in lower cellular risk with the coated particle. Mechanistically, this effect is plausible due to several factors: (i) colloidal stabilization and protection against curcumin degradation; (ii) reduction of immediate drug release; and (iii) modulation of particle–membrane interactions, as chitosan organizes the interface and can reduce uncontrolled interactions with the cell bilayer. These observations indicate that the coating increases stability and delays drug release compared to uncoated liposomes, which likely explains the higher CC_50_ observed for Lipo-CUR-Chi.^47–49^

### 3.6 Evaluation of *In Vitro* Activity in Promastigotes

The antipromastigote activity of CUR demonstrated a clear species-dependent profile, as evidenced by both the concentration–response curves and IC_50_ values (Figure 4, Table 5). Free CUR exhibited a gradual, concentration-dependent reduction in parasite viability, with a more pronounced effect against *L*. *amazonensis* compared to *L*. *infantum*.

**Figure 4:**
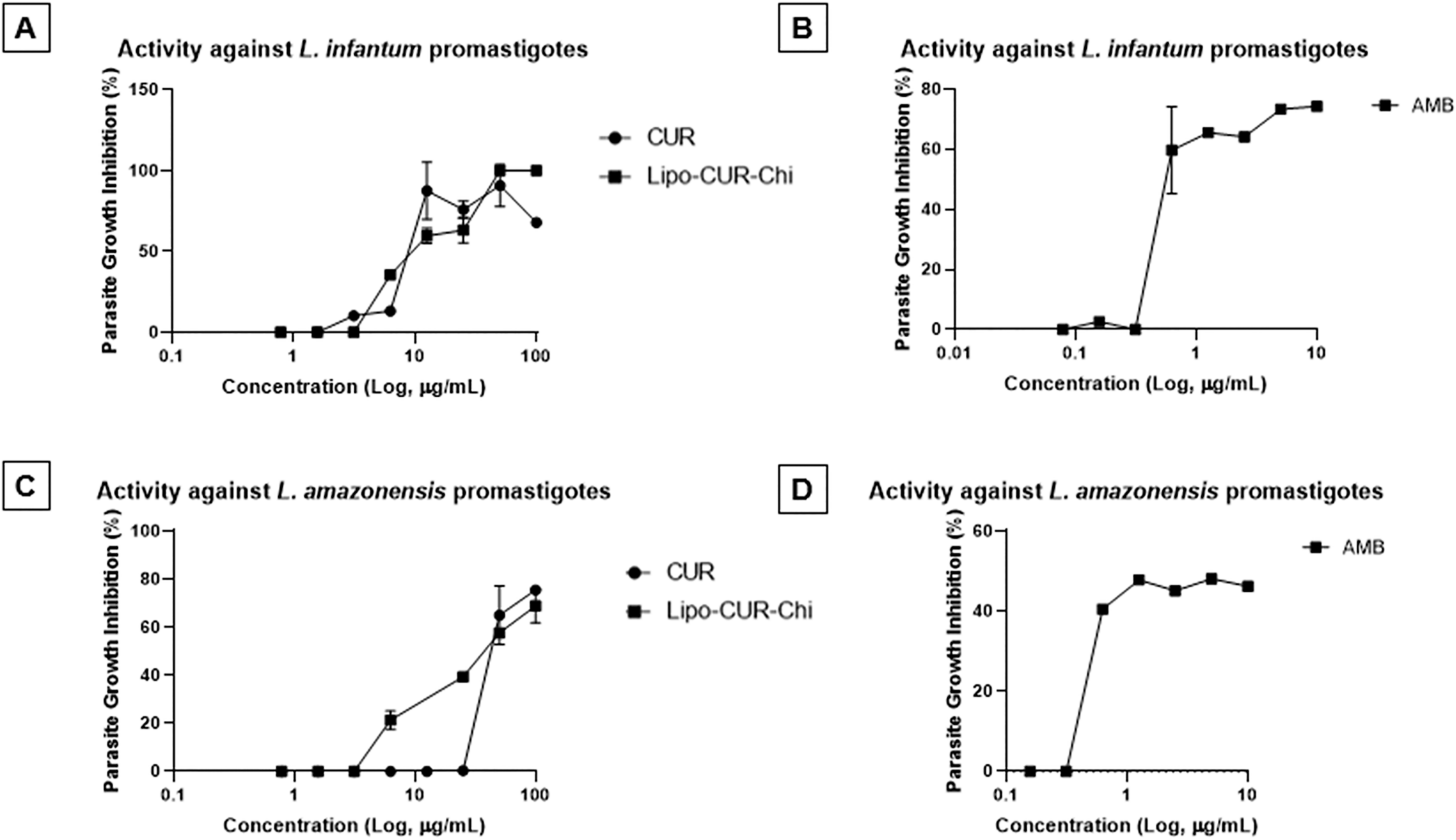
Inhibitory effect of the tested formulations against promastigote forms. Parasite viability (%) was evaluated across increasing concentrations, demonstrating a concentration-dependent reduction in viability.

**Table 5:** IC_50_ Values on Promastigotes, CC_50_ on RAW 264.7 Macrophages and Selectivity Index (SI) of the Different Compounds Tested against *L*. *infantum* and *L*. *amazonensis* Expressing β-Galactosidase^a^

*L*. *infantum*			
compound	IC_50_ on promastigotes	CC_50_ over RAW	SI (promastigotes)
μg/mL			
CUR	7.9 ± 0.6	3.7 ± 0.1	0.5
Lipo-Chi	ND	ND	ND
Lipo-CUR-Chi	11.5 ± 1.6	12.7 ± 0.1	1.1
AmB	0.6 ± 0	8.5 ± 0	14.2
*L*. *amazonensis*			
CUR	43.5 ± 0.4	3.7 ± 0.1	0.09
Lipo-Chi	ND	ND	ND
Lipo-CUR-Chi	16.7 ± 3.5	12.7 ± 0.1	0.8
AmB	0.6 ± 0	8.5 ± 0	14.2

a CUR: Curcumin; Lipo-Chi: Chitosan-coated liposomes; ND: Not determined; Lipo-CUR-Chi: Liposome containing curcumin coated with chitosan; AmB: Amphotericin B; IC_50_: Concentration of compound that reduces parasitic growth by 50%; CC_50_: Concentration of compound that inhibits the viability of RAW 264.7 macrophages by 50%; SI: CC_50_ of the compound on RAW 264.7 macrophages divided by the IC_50_ of the compound on promastigotes. The assays were performed in duplicate for each concentration of the compound under analysis, and the IC_50_ and CC_50_ values correspond to the mean ± standard deviation of two technical replicates. The IC_50_ and CC_50_ values were calculated by nonlinear regression analysis using GraphPAD 8.

For *L*. *infantum*, CUR showed limited efficacy, as reflected by a relatively high IC_50_ value (43.5 ± 0.4 μg/mL) and a low selectivity index (SI = 0.09). This profile is consistent with the shallow slope observed in the inhibition curve, indicating reduced sensitivity of this species and limited antipromastigote activity. In contrast, *L*. *amazonensis* displayed greater susceptibility to CUR, with a lower IC_50_ (7.9 ± 0.6 μg/mL) and a modest improvement in selectivity (SI = 0.5), corroborated by a more pronounced decline in viability across increasing concentrations.

Amphotericin B, used as a positive control, exhibited a steep and consistent inhibition profile in the curves, achieving near-complete parasite inhibition at low concentrations. This is reflected in its low IC_50_ (0.6 μg/mL) and high selectivity index (SI = 14.2), confirming both its high potency and the reliability of the assay conditions.

In contrast to free CUR, Lipo-CUR-Chi demonstrated a markedly enhanced antipromastigote effect, as evidenced by a steeper and more pronounced concentration–response profile for both species. For *L*. *infantum*, the nanoformulation significantly improved efficacy, reducing the IC_50_ to 16.7 ± 3.5 μg/mL and increasing the SI to 0.8. This improvement is consistent with the shift of the inhibition curve toward lower concentrations, indicating increased potency and improved interaction with the parasite.

A more pronounced enhancement was observed for *L*. *amazonensis*, where Lipo-CUR-Chi produced a stronger inhibitory effect across the entire concentration range. The IC_50_ values ranged from 6.2 ± 0.1 to 11.5 ± 1.6 μg/mL, with corresponding selectivity indices between 1.1 and 1.5. As expected, Lipo-Chi did not exhibit activity under the tested conditions.

The comparative analysis demonstrates that the response to the compounds was species-dependent. Free curcumin showed low efficacy against *L*. *infantum* but performed better against *L*. *amazonensis*. This behavior has been reported in the literature, where differences among *Leishmania* species influence susceptibility to phenolic compounds due to variations in oxidative metabolism and cell membrane composition.^50–52^

Encapsulation of curcumin in chitosan-coated liposomes resulted in a significant improvement in selectivity and potency against *L*. *amazonensis* compared to free curcumin.^53^ These results corroborate previous studies showing that liposomal encapsulation increases curcumin stability and bioavailability, in addition to protecting the compound from rapid degradation in biological media.^51,54^ In contrast, against *L*. *infantum*, the effect of encapsulation was more modest, indicating that this species may present additional physiological barriers to the action of nanostructured curcumin.^54^

Compared to the therapeutic standard, amphotericin B remained more potent and selective against both species.^55^ Nevertheless, the results with Lipo-CUR-Chi, particularly against *L*. *amazonensis*, indicate promising potential for use in alternative or adjuvant formulations for leishmaniasis treatment, with lower toxicity than conventional drugs.

Evaluation of drug activity against the promastigote form of *Leishmania* sp. is important as an initial screening step, as it allows the identification of compounds with leishmanicidal potential before testing intracellular forms.^56^ Although this parasite form is extracellular and does not directly represent the clinical phase of infection, its sensitivity to the drug provides preliminary information on compound efficacy, contributing to the selection of promising formulations for subsequent testing in amastigotes, which reflect the intracellular phase relevant for leishmaniasis therapy.^57^

### 3.7 Evaluation of *In Vitro* Activity in Amastigotes

In tests using intracellular amastigote forms of *L*. *infantum* expressing β-galactosidase (Table 6), CUR presented an IC_50_ of 2.8 ± 0.3 μg/mL, with a CC_50_ of 3.7 ± 0.1 μg/mL in macrophages, resulting in an SI of 1.3. In contrast, Lipo-CUR-Chi showed markedly improved activity (Figure 5), with an IC_50_ of <0.78 μg/mL and a CC_50_ of 12.7 ± 0.1 μg/mL, resulting in a substantially higher SI (16.2), indicating enhanced selectivity and reduced cytotoxicity. Amphotericin B, used as the reference drug, presented the greatest potency, with an IC_50_ of 0.13 μg/mL and an SI of 65.4, as also reflected by the steep dose–response curve (Figure 5).

**Figure 5:**
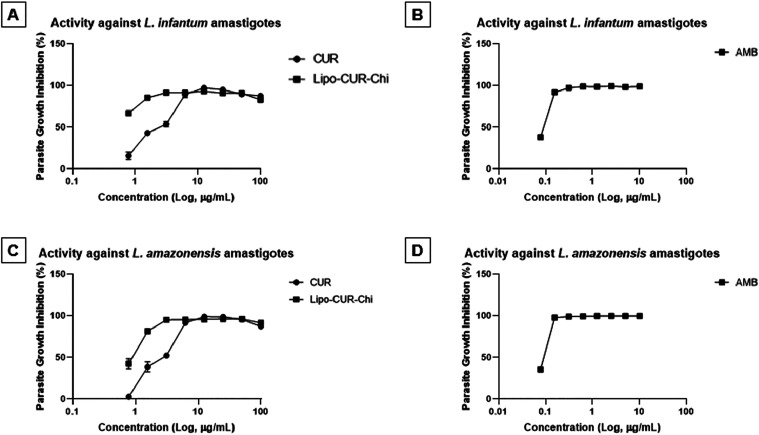
Dose–response curves showing the activity against intracellular amastigotes of Leishmania infantum infection. (A) CUR and Lipo-CUR-Chi. (B) AmB. Data are expressed as percentage of inhibition relative to untreated controls, demonstrating a concentration-dependent effect, with enhanced activity for the nanoformulation compared to free CUR.

**Table 6:** IC_50_ Values on Amastigotes, CC_50_ on RAW 264.7 Macrophages and Selectivity Index (SI) of the Different Compounds Tested against *L*. *infantum* and *L*. *amazonensis* Expressing β-Galactosidase^a^

*L*. *infantum*			
compound	IC_50_ on amastigotes	CC_50_ over RAW	SI (amastigotes)
μg/mL			
CUR	2.8 ± 0.3	3.7 ± 0.1	1.3
Lipo-Chi	ND	ND	ND
Lipo-CUR-Chi	<0.78	12.7 ± 0.1	>16.3
AmB	0.13 ± 0	8.5 ± 0	65.4
*L*. *amazonensis*			
CUR	2.5 ± 0.1	3.7 ± 0.1	1.5
Lipo-Chi	ND	ND	ND
Lipo-CUR-Chi	1.4 ± 0	12.7 ± 0.1	9.1
AmB	0.13 ± 0	8.5 ± 0	65.4

a CUR: Curcumin; Lipo-Chi: Chitosan-coated liposomes; ND: Not determined; Lipo-CUR-Chi: Liposome containing curcumin coated with chitosan; AmB: Amphotericin B; IC_50_: Concentration of compound that reduces parasitic growth by 50%; CC_50_: Concentration of compound that inhibits the viability of RAW 264.7 macrophages by 50%; SI: CC_50_ of the compound on RAW 264.7 macrophages divided by the IC_50_ of the compound on amastigotes. The assays were performed in duplicate for each concentration of the compound under analysis, and the IC_50_ and CC_50_ values correspond to the mean ± standard deviation of two technical replicates. The IC_50_ and CC_50_ values were calculated by nonlinear regression analysis using GraphPAD 8.

In the evaluation against *L*. *amazonensis*, CUR presented an IC_50_ of 2.5 ± 0.1 μg/mL, with low selectivity (SI = 1.5). Lipo-CUR-Chi again demonstrated improved activity, with an IC_50_ of 1.4 ± 0.2 μg/mL and an SI of 9.1. Amphotericin B remained the most effective compound, with an IC_50_ of 0.13 μg/mL and high selectivity (SI = 65.4). These findings are consistent with the dose-dependent inhibition profiles observed in Figure 5, reinforcing the superior performance of the nanoformulation compared to free CUR. As expected, Lipo-Chi did not exhibit activity under the tested conditions.

The comparative analysis of the compounds revealed significant differences in the susceptibility of *L*. *infantum* and *L*. *amazonensis*, confirming that the response to treatment is species-dependent. Against *L*. *infantum*, free CUR showed moderate activity against amastigotes but low selectivity (SI = 1.3). This result aligns with recent studies demonstrating the limited efficacy of free curcumin due to low solubility, stability, and bioavailability.^58^

Encapsulation of CUR in chitosan-coated liposomes resulted in a marked increase in potency and selectivity against *L*. *infantum*, greatly surpassing free curcumin. This behavior corroborates reports highlighting the ability of liposomal nanoformulations to enhance curcumin stability, prolong its half-life, and optimize intracellular delivery.^59^ Moreover, the presence of chitosan in the coating enhances interactions with membranes and specific receptors, a mechanism also described in mannosylated nanoparticles containing curcumin, which showed significant reductions in parasitic load in visceral leishmaniasis models.^60^

In the evaluation against *L*. *amazonensis*, free CUR exhibited an IC_50_ of 2.5 μg/mL in amastigotes, with low selectivity (SI = 1.5). In contrast, the Lipo-CUR-Chi formulation demonstrated superior performance, with an IC_50_ of 1.4 μg/mL and SI = 9.1, evidencing higher selectivity against this species. These findings reinforce that combining curcumin with chitosan-coated liposomes is an effective strategy to increase both potency and selectivity, particularly against amastigotes, the primary form responsible for maintaining infection in vertebrate hosts.^61^

Once again, amphotericin B, used as a control, remained the most potent and selective compound against both species, as well established in the literature.^62^ Nevertheless, the results obtained with Lipo-CUR-Chi are promising, especially considering the potential to reduce the toxic effects characteristic of conventional treatments, since selectivity indices significantly higher than those of free curcumin were achieved for both *L*. *infantum* and *L*. *amazonensis*.

## 4 Conclusion

The curcumin formulation in chitosan-coated liposomes exhibited stable physicochemical properties, high encapsulation efficiency, and an appropriate release profile, resulting in enhanced antimicrobial and antiparasitic activity compared to free curcumin. The formulation showed bactericidal effects against different pathogenic *E*. *coli* strains, including biofilm inhibition, and improved activity against promastigote and amastigote forms of *Leishmania* spp. In macrophage assays, the formulation demonstrated a more favorable selectivity profile than free curcumin and reduced cytotoxic effects when compared with conventional drugs such as amphotericin B.

Nevertheless, although the results indicate a promising therapeutic profile, further optimization is required to ensure efficacy at strictly noncytotoxic concentrations. In vivo studies will be essential to confirm the therapeutic potential of this nanotechnological platform and to explore its applicability, including its use as an adjuvant in combination therapies for bacterial infections and neglected parasitic diseases such as leishmaniasis.
